# Applying Expression Profile Similarity for Discovery of Patient-Specific Functional Mutations

**DOI:** 10.3390/ht7010006

**Published:** 2018-02-22

**Authors:** Guofeng Meng

**Affiliations:** BT science Inc., No. 24, Tang’an Road, Shanghai 201203, China; menggf@gmail.com

**Keywords:** driver mutation, expression similarity, breast cancer

## Abstract

The progress of cancer genome sequencing projects yields unprecedented information of mutations for numerous patients. However, the complexity of mutation profiles of cancer patients hinders the further understanding to mechanisms of oncogenesis. One basic question is how to find mutations with functional impacts. In this work, we introduce a computational method to predict functional somatic mutations of each patient by integrating mutation recurrence with expression profile similarity. With this method, the functional mutations are determined by checking the mutation enrichment among a group of patients with similar expression profiles. We applied this method to three cancer types and identified the functional mutations. Comparison of the predictions for three cancer types suggested that most of the functional mutations were cancer-type-specific with one exception to *p53*. By checking predicted results, we found that our method effectively filtered non-functional mutations resulting from large protein sizes. In addition, this method can also perform functional annotation to each patient to describe their association with signalling pathways or biological processes. In breast cancer, we predicted “cell adhesion” and other terms to be significantly associated with oncogenesis.

## 1. Introduction

Cancer arises from accumulations of somatic mutations and other genetic alterations, which leads to abnormal cell proliferation [1]. With the progress of cancer genome sequencing projects, such as the Cancer Genome Atlas (TCGA) project, mutation information for numerous patients are becoming publicly available [2,3,4], which provides the foundation to uncover mechanisms of oncogenesis. However, cancer patients usually carry an average of 33 to 66 mutations in the protein coding regions and those mutations are supposed to take unequal roles in oncogenesis [5,6]. It remains a great challenge to distinguish the functional mutations that give cells with growth advantages [7], from the ones with non-crucial roles to oncogenesis.

Many computational tools have been developed to predict the functional mutations. One popular strategy is to find recurrently mutated genes. Genes with higher mutation frequency are supposed to take an essential role in oncogenesis [6,8,9,10,11]. To improve the accuracy, most computational tools also consider background mutation rates and protein sizes in discovery of recurrent mutations [12,13,14]. Another strategy is mutual exclusivity, which is based on an assumption that if one gene is mutated in a patient, other members acting in the same signaling pathway are less likely to be mutated [15]. Using mutual exclusivity, members of signalling pathways are investigated for their coverage across a large number of patients while not co-mutated in the same patients [16,17,18,19]. HotNet2, a network approach is applied to identify significantly mutated groups of interacting genes across pathways and protein complexes [20]. Besides of functional annotations, expression information is also used in driver mutation discovery. One popular application is to find functional copy number variations (CNVs) by identifying the differentially expressed genes located in the CNV regions [21]. For somatic mutations, correlation of gene expression profiles is also used to identify the functional mutations based on the assumption that somatic mutations will result in altered expression of their downstream targets [22].

Somatic mutations in coding regions are usually supposed to involve oncogenesis by affecting the activities of proteins associated with cell proliferation [23]. However, somatic mutations at different sites have different effects on protein activities. One solution is to study the stability of protein structures after mutations. Amino acid changes in the protein sequences can either stabilize, destabilize or have no effect on protein structures. Methods based on this strategy calculate the protein folding free energy, which is used to evaluate the protein structural stability [24,25,26]. However, those methods still face the problems of establishing the direct connections between protein structures and activities.

In this work, we introduce a method to recover functional somatic mutations for each of the studied patients by integrating mutation recurrence and expression profile similarity among patients. This method is based on two assumptions: (1) functional mutations will lead to altered gene expression which reflects consequences of somatic gene mutations; (2) patients with similar expression profiles are more likely to carry functional mutations to the same genes. For each patient, we can find a group of patients that have similar expression profiles to him/her and those patients are called neighboring patients. Mutations of the studied patient are evaluated for their enrichment among all the neighboring patients. Mutations with enough enrichment are predicted to be functional mutations. The functional mutations of all patients can be recovered by repeating this process. This method also performs function annotation analysis to mutated genes in neighboring patients so that each patient can be assigned with some functional terms to indicate the association with signalling pathways or biological processes. As applications, we applied this method to three cancer data sets and identified the functional mutations for three types of cancers respectively.

## 2. Materials and Methods

### 2.1. Dataset and Their Pre-processing

In this work, we focus on prediction of functional somatic mutations, including missense mutation, nonsense mutation and frame-shift. All the mutation and expression data are downloaded from TCGA project (by May 2013) at level two, with which the expression data have been normalized within samples and that the somatic mutations have also been annotated in protein coding genes by the pilot of TCGA project. We filtered those patients with only mutation or expression data for next-step analysis.

### 2.2. Expression Biomarkers

To describe the expression profile similarity of patients, we firstly determine a group of genes or probes as expression biomarkers by selecting the top 2000 genes or probes with the most expression variances among all the patients. These biomarkers are supposed to better reflect the expression consequences of gene mutations. The expression profile of one patient is described as a vector with the expression values of biomarkers as the elements.

### 2.3. Neighboring Patients: Patients with Similar Expression Profiles

The similarity of expression profiles of the patients was measured by using Pearson’s correlation *r*. Conversely, the distances between patients could be described by 1−r. For each patient, we could find his/her neighboring patients by selecting *n* patients with most significant positive correlation values at a minimum *r* cutoff (e.g., r>0.6), where *n* ranged from 5 to 30.

### 2.4. Mutation Network

The synergy of functional mutated genes were evaluated by checking their cooccurence in the patients. We calculated the synergy score for gene *a* and *b* as:

$$s_{A,B}=\frac{\left||A\cup B|\right|}{max(||A||,||B||),}$$

where ||A|| and ||B|| are the set sizes of mutated patients for gene a and b.

The functional mutated genes with co-occurrence were connected by weighted edges to build a mutation network, where synery scores defined the connection strength. We also used node sizes to indicate the functional mutation frequency in the breast cancer patients.

## 3. Results

### 3.1. Association of Mutation and Expression Profiles

**Figure 1:**
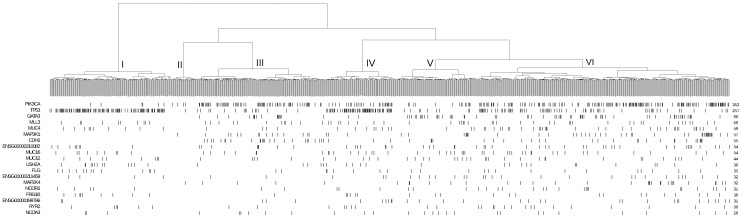
Hierarchical clustering and the mutation distribution of gene.

As an exemplary illustration, we checked the association between somatic mutations and the expression profile similarity by investigating 516 breast invasive cancer patients. We selected 2000 probes with most expression variances as the biomarkers and performed hierarchical clustering to the patients. Then we checked the distribution of mutated genes on the hierarchical tree. One example is CDH1 gene, which is mutated in 35 out of 516 patients. As showed in Figure 1, patients with CDH1 gene mutation were not randomly distributed but preferentially clustered together. To quantify this, we classified the patients into six groups based on the structure of hierarchical clustering tree. Among groups, the frequency of CHD1 mutations varied greatly. In Cluster IV, we observed 22 out of 120 patients to carry CDH1 mutations, which was also the most enriched group. Fisher’s exact test indicated the significance of this enrichment to be $p<4.4\times 10^{-8}$. In cluster VI, there were 6 out of 70 patients to carry CDH1 mutation without statistical significance. For cluster I and V, there was even no patient with CDH1 mutation. We also checked the mutation distribution of other genes and observed non-random distribution in the expression subclusters in nearly all the test cases.

These results suggest that patients with a similar set of mutated genes are more likely to have similar expression profiles, which can be explained by the fact that gene mutations would directly or indirectly affect transcription of their downstream targets [22,27]. Based on this observation, we propose that genes affected by functional mutations can be identified by checking their enrichment in patients with similar expression profiles.

### 3.2. Pipeline to Find Functional Mutations

In this work, we propose a computational method to infer the functional mutations for each studied patient. This method is based on two assumptions that, (1) genes carrying functional mutations directly or indirectly lead to altered expression of their downstream target genes; (2) patients with the same functional mutations will share similar expression profiles. Thus, for each mutated gene from one patient, we can check its mutation enrichment in the neighboring patients. The impact of gene mutation to studied patients can be described by a *p*-value based on statistical frameworks.

**Figure 2:**
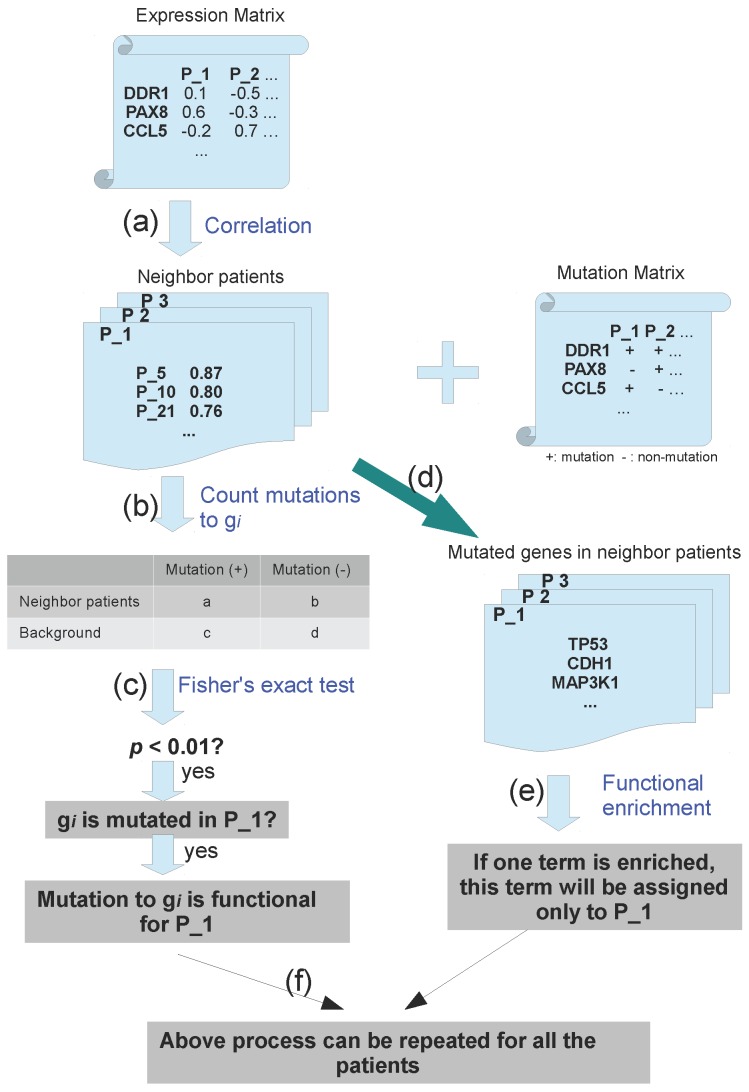
Pipeline to predict functional mutations and terms (see main text for detailed description).

The whole process is described in Figure 2. This method requires two matrices as input: one expression matrix and one binary mutation matrix. As described in Section 2, the expression matrix is explored to determine the neighbor patients by checking its expression similarity. For patient $P_{1}$ with a mutation to gene $g_{i}$, his/her neighboring patients are selected by choosing *n* patients (including $P_{1}$) with the most similar expression profiles with $P_{1}$, where *n* ranged from 5 to 30 (Figure 2a). Then, the patient number with mutation to gene $g_{i}$ is counted in all the neighbors of $P_{1}$ (Figure 2b). Based on randomly simulation, in which the neighbor patients are randomly selected, the enrichment of mutations to $g_{i}$ among neighbor patients is evaluated by Fisher’s exactly test. A *p*-value is assigned to studied patient to describe its mutation enrichment to $g_{i}$ (Figure 2c), which also indicates its functional importance. If the *p*-value is significant enough (e.g., 0.01), mutation to gene $g_{i}$ in patient $P_{1}$ will be treated as functional mutation of only patient $P_{1}$. The same process can be repeated for other mutated genes and patients one by one (Figure 2f).

With our method, we also check the functional annotation for each studied patient by enrichment analysis to all the mutated genes in neighboring patients. The functional annotation is described by Gene ontology (GO) or Kyoto Encyclopedia of Genes and Genomes (KEGG) terms. In this step, all the mutated genes from patient $P_{1}$ and his/her neighboring patients (Figure 2d) are checked for enriched functional terms by comparing to the background occurrences with Fisher’s exact test (Figure 2e). If a term is significantly enriched (e.g., p<0.01), it will be assigned to patient $P_{1}$ as functional annotation. This term indicates the functional consequences of gene mutations in patient $P_{1}$. For the other patients, the same process can be repeated to find their associated functional terms (Figure 2f).

### 3.3. Application to Breast Cancer

As an illustration, we applied our method to 516 breast cancer patients from the TCGA project. The functional mutations were chosen at a cutoff of p<0.01. In Table 1, we show 10 genes with the most functional mutation recurrences in breast cancer. For all the genes, only part of their mutations is predicted to be functional. Among them, PIK3CA and TP53 genes were especially recurrently mutated based on our prediction. PIK3CA gene was observed to be mutated in 175 breast cancer patients and 123 of them were confirmed to be functional (about 70.3%), which made PIK3CA to be the gene with the most confirmed mutations, even though it was not the most somatic mutated genes in breast cancer. Another gene was TP53 with functional mutations in 107 patients, about 56.9% of observed somatic mutations. There were also other functional mutated genes, such as MAP3K1 [28], CDH1 [29] and GATA3 genes. Compared to PIK3CA and TP53, relatively fewer functional mutations were observed. However, many of them have been widely reported for their critical roles in cancer, which indicated their important roles but in fewer patients. To sum up, we observed 408 of 516 breast cancer patients to carry at least one of these 10 predicted driver mutations, covering about 79% of all the patients.

In Table 1, we also show the driver cancer genes predicted by MutSigCV [10], MUSIC [30] and drGAP [31]. We found that 8 out of 10 functional mutated genes were predicted to be driver cancer genes by at least one of three tools at a cutoff of p<0.05. Two transcriptional regulators, including RUNX1 and CTCF, were not identified as driver cancer genes. By checking the published literatures, we found both genes to be related with breast cancer. RUNX1 is implicated in proliferation control of breast cancer [32,33]. CTCF is reported to be associated with resistance to apoptosis in breast cancer [34]. Overall, the predicted functional cancer genes are all related with breast cancer.

Further, we tested whether the predicted mutations were functional. We checked the genes with recurrent somatic mutations while less predicted to be functional. One example was the TTN gene. It is mutated in 90 patients, ranked as the third most recurrent mutated gene in breast cancer. However, we only predicted three of them to be functional. TTN protein is a component of vertebrate striated muscle [35]. By searching literature, we did not find any reports to support TTN to take critical role in cancer. By checking its protein sequence, we found that it was very long with a length of 27,000 to 33,000 amino acids [36], indicating a recurrence bias of its mutations. This prediction was also confirmed by the analysis results with MutSig, which considers both the protein size and background mutation rates into evaluation [12]. Similar results are observed with other genes, such as MUC6 with mutations in 57 patients while predicted to be functional in only two patients. Even though our pipeline did not consider the protein size, it successfully identified fake recurrent mutations resulting from large protein sizes. These results provide evidence to support the specificity of our method to recover functional mutations.

### 3.4. Mutation Types

**Figure 3:**
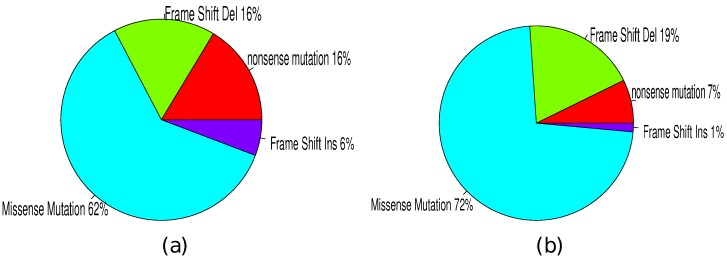
Mutation type differences between functional (**a**) and non-functional TP53 mutations (**b**).

Based on mutation types, somatic mutation can be categorized into missense mutation, nonsense mutation, silent mutation, frame shift insertion and frame shift deletion. Taking TP53 as an example, we checked the mutation types between two groups of TP53 mutations: (1) functional TP53 mutation as identified by our method and (2) non-functional TP53 mutations. As showed in Figure 3, most of the mutations were missense mutations (∼65 %). Statistical test did not support any significance difference between two groups of patients. However, nonsense mutation and frame shift insert types had great ratio differences between two groups. 16% of functional TP53 mutations were observed to be nonsense mutations while only 7% of non-functional TP53 mutations. The significance based on Fisher’s exact test was at p<0.03. A similar result was observed with frame shift insertion, which was 6% of functional mutations and 1% of non-functional mutations (p<0.12). The enrichment of those types of mutations can be explained by the fact that nonsense mutations and frame shift insertions are more likely to disrupt protein function than missense mutation. We also checked other recurrently mutated genes and similar results were observed. These results suggest that predicted functional mutations are more likely to influence the protein function.

### 3.5. Functional Mutated Genes in Other Cancers

**Figure 4:**
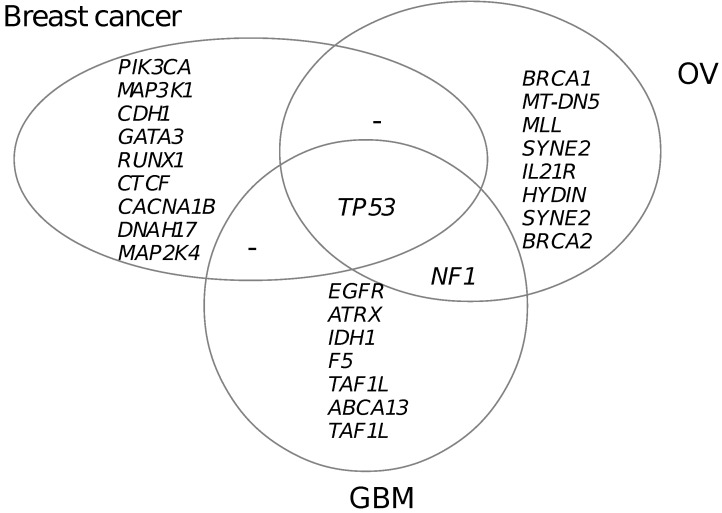
Predicted genes with recurrent functional mutation for three cancers. OV: Ovarian serous cystadenocarcinoma; GBM: Glioblastoma multiforme.

Besides breast cancer, we also applied our method to ovarian serous cystadenocarcinoma (OV) and glioblastoma multiforme (GBM) using data from the TCGA project. Figure 4 shows the predicted genes carrying functional mutations in three cancers. By checking the published literature, we found that most of the genes have been implicated for important roles in oncogenesis [6]. However, there are mutation preferences among three cancer types. As showed in Figure 4, only TP53 gene is shared by all three cancer types and NF1 gene is shared by OV and GBM. Other genes are preferentially mutated in one cancer type, which can be called cancer type-specific mutations. These cancer type-specific mutations had higher mutation recurrences and were supposed to take more essential roles in the oncogenesis of a specific cancer type.

Cancer-specific mutations are not necessary to indicate non-function in other cancers. One example is BRCA1 gene. BRCA1 was predicted to be mutated in 17 out of 441 patients for OV, ranking as the third most recurrently mutated gene. Based on our definition, BRCA1 is supposed to be OV-specific. However, we also observed functional mutations to BRCA1 in breast cancer. The roles of BRCA1 in the oncogenesis of breast cancer have been widely reported [37] and researches even suggest that patients with BRCA1 mutations have a possibility of 50%–80% to develop breast cancer before the age of 70 [38]. However, mutations to BRCA1 is not supposed to be the most important causal mutation only due to the relatively low mutation recurrence in breast cancers, which was at 6 out of 516 patients.

### 3.6. Functional Association of Functional Mutations

**Figure 5:**
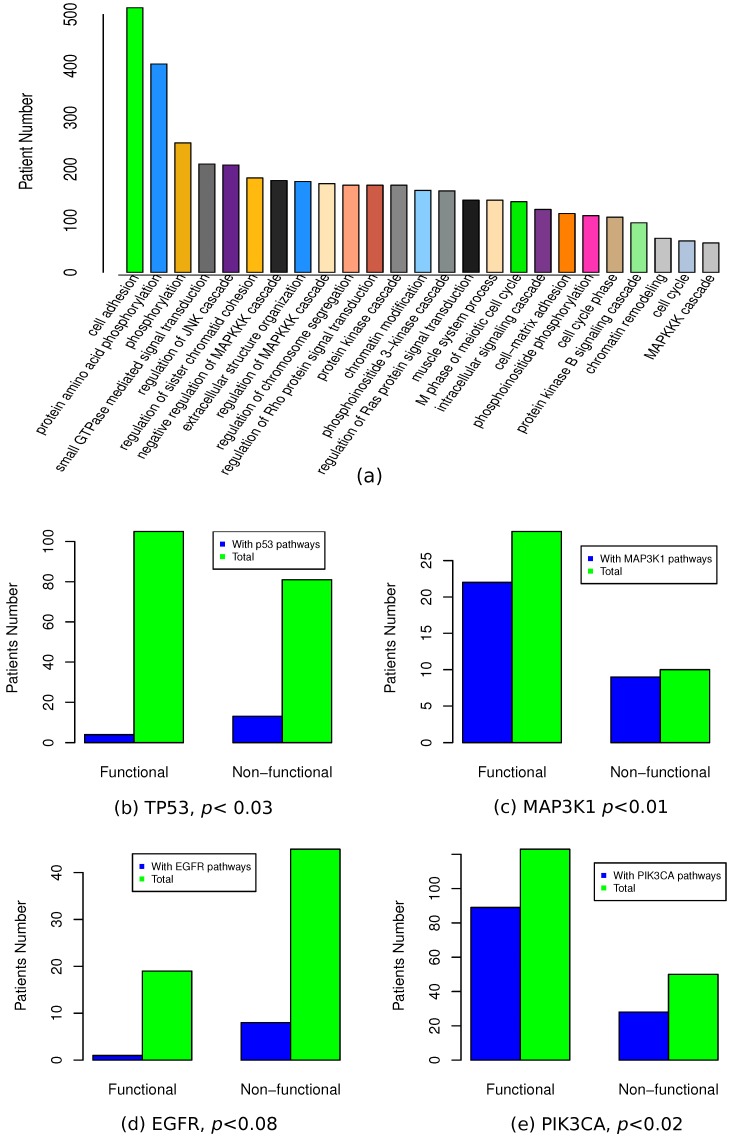
Functional enrichment to functional mutations. (**a**) Gene ontology (GO) terms enriched with breast cancer patients; (**b**-**e**) differences between patients with or without predicted functional mutations.

The patients with the similar expression profiles are supposed to carry the mutations to the same pathway. Consistent with the concept of mutual exclusivity, the affected pathways can be recovered by enrichment analysis to patients with similar expression profiles. Therefore, we performed functional annotations for each of studied patients by functional enrichment analysis to all the mutated genes of neighboring patients. At a cutoff of p<0.01, we observed some terms associated with a large proportion of breast cancer patients (see Figure 5a). One example was the term “cell adhesion”, which was associated with 99.4% of patients. Similarly, other extracellular matrix (ECM) related terms such as “extracellular structure organization” and “cell–matrix adhesion”, were also associated with 34.1% and 22.1% of patients respectively. These observations are consistent with reports about ECM for its critical roles in cancer [39,40]. This result also suggests ECM to be one potential target to develop anticancer drugs. Above, we reported recurrently mutated genes, such as PIK3CA and MAP3K1. Functional annotation supported their roles by enriched GO terms, such as “phosphoinositide 3-kinase (PI3K) cascade” (30.6% of patients) and “MAPKKK cascade” (34.5% of the patients). Considering the fact that PIK3CA and MAP3K1 were ranked as the most recurrently mutated genes, PI3K signalling pathway and MAPKKK cascade pathway were supposed to be essential pathways during the oncogenesis of breast cancer. Besides these pathways, we also observed enriched terms such as “JNK cascade” and “cell cycle phase”, which had been reported to be related to oncogenesis of cancers [1].

Based on textbook knowledge and published literature, we could assign some genes to some well-studied pathways or biological processes. For example, TP53 is involved in the p53 mediated DNA damage response [41]; MAP3K1 in the MAPKKK cascade pathway [42]. These terms could be used to evaluate functional differences between patients predicted with or without functional mutations. Taking TP53 mutation of breast cancer as an example, we checked the number of patients with p53 associated term “DNA damage response, signal transduction by p53 class mediator”. As showed in Figure 5b, we observed 6 out of 107 patients with predicted functional TP53 mutation to be annotated with this term while 12 out of 81 non-functional TP53 mutation carriers were annotated with this term. Fisher’s exact test indicated a significant ratio difference at p<0.03. Besides of breast cancer, we also performed the same evaluation with OV and GBM. Similar results were observed with TP53 mutations in OV. In GBM, we did not observe significant differences, which might be due to the limited number of patients with TP53 mutations, which was only 65 patients. In a way, these results suggest that patients with functional TP53 mutations are less likely to carry other mutations to the members of TP53 associated pathways. In breast cancer, we also checked MAP3K1 mutation with the similar methods for “MAPKKK cascade” term (see Figure 5c). Like TP53, we observed that patients with functional MAP3K1 mutations were less likely to carry the mutations to other components of MAPKKK cascade pathways (p<0.01). In GBM, we checked another well-known gene EGFR for that it involved in “epidermal growth factor receptor signaling pathway” (see Figure 5d). Similarly, other genes from EGFR signaling pathways were less likely to be mutated in patients with functional EGFR mutations (p<0.08). In summary, all above examples were consistent with the popular assumption of mutual exclusivity [15]. However, one contrary example was also observed with predicted PIK3CA mutations in breast cancer (see Figure 5e). By checking PIK3CA associated term “phosphoinositide 3-kinase cascade”, more members of PI3K cascade pathway were observed with predicted functional PIK3CA mutation carriers (p<0.02), which was consistent with previous reports about interactions of PIK3CA with other oncogenes [43,44]. This result indicates that mutual exclusivity is not applicable to PIK3CA mutations.

### 3.7. Mutation Network

**Figure 6:**
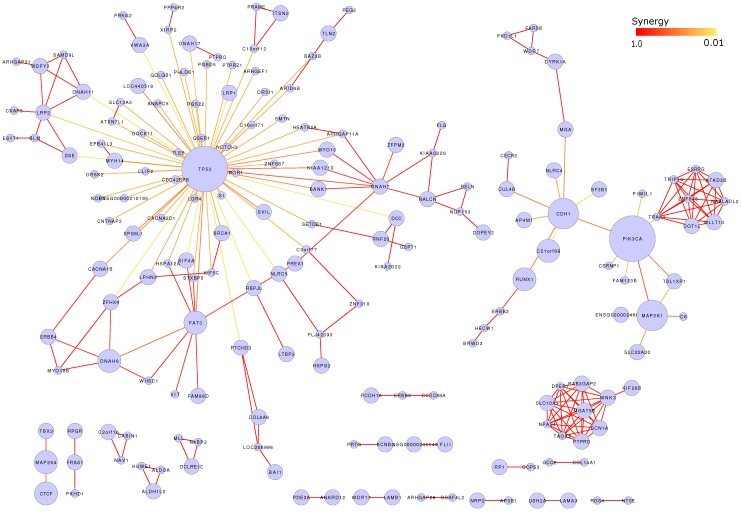
Synergistic network of functional mutated genes.

Based on predicted functional mutations, we computed the synergy of mutated genes in breast cancer. We connected the genes with functional mutations in shared patients and displayed those genes as a network (see Figure 6). Each node was one mutated gene, with node size to indicate its mutation recurrences. We also displayed the synergy strength by colored edges and dark red indicated stronger synergy. In this network, we observe genes, such as TP53, PIK3CA1, MAP3K1 and CDH1, to take hub roles by connecting to other genes. By checking number of connection, we find different genes to take unequal synergy with other genes. For example, 149 genes are connected to TP53 while only seven genes connect to PIK3CA and five genes connect to MAPK3K1. This observation suggests that TP53 can easily have synergy with many other genes. This may explain why TP53 mutation is always functional in multiple cancer types. For other recurrently mutated genes, such as PIK3A, MAP3K1 and CDH1, they nearly have no connection with TP53, indicating their functional independence to TP53. However, interplays are observed among themselves. This is especially true for PIK3CA, which has strong synergy with both MAP3K1 ($p<9.1\times 10^{-22}$) and CDH1 ($p<3.7\times 10^{-15}$).

In this network, we find some sub-networks, where their nodes are intensively connected with each other but less or no connection to the hub genes. One example was a sub-cluster of 10 genes, including WNK3, SCN1A, PTPRD, TAOK3, NPAS4, SLC10A3, DPEP1, RAB3GAIP2, MGAT5B and KIF26B. By searching published reports, we found the potential interactions between WNK3 and SLC10A3 [45]. For other genes, we are still not clear their interactions. However, their synergy is supposed to be necessary for some breast cancer patients.

## 4. Discussion

In this work, we propose a computational method for discovery of functional mutation for each studied cancer patient. One basic question is what are functional mutations to cancer. Indeed, different tools have quite different definitions. In the context of this work, functional mutations are mutations that directly or indirectly lead to altered expression of many genes; they are the main causal reasons for observed expression patterns of patients. Thus, patients with the same functional mutations are supposed to have similar expression profiles. Following this definition, we transfer identification of functional mutations of one patient to enrichment analysis to mutations in its neighboring patients. This strategy can be called “guilt by association”, as we have done in our previous work [46]. By checking the published literature, we can find evidence to support this definition. For example, Lengerod et al [47,48] observed characteristic gene expression patterns in patients carrying TP53 mutations. Similar results are available for other cancer genes, such as BRAF in melanoma [27], BRCA1 [49] and PIK3CA in breast cancer [50]. In this work, we also observed patients carrying the same mutation to be clustered together.

The functional mutations are identified by checking their mutation recurrence in patients with similar expression profiles. The complexity of mutation profiles in cancer patients makes it impossible to cluster all the patients with the same mutation into one cluster. Most of the pattern classification methods, such as k-means, support vector machine (SVM), do not consider the sub-structure of samples, which cannot ensure each patient to have the optimal neighbors. With our method, we used a simple method to find neighbors for every patient one by one, which makes it good at discovering the patterns of inner cliques. Utilization of statistical significance also makes this method more robust.

With our prediction, some somatic mutations are not predicted to be functional. It is natural to raise the questions what those non-functional mutations are. One explanation is that non-functional mutations fail to affect the protein function. As an evaluation, we checked protein structure stability between mutated genes with or without predicted function. The folding free energy was calculated for each mutation by PoPMuSic2.1 [51]. The energy differences were further evaluated for four examples: TP53, PIK3CA, MAP3K1 and EGFR. However, we only observed a significant difference in case of TP53 at p<0.03, which suggested functional mutations to TP53 were more affect its protein structure. For others, no significant differences were observed, which may result from the low accuracy of computational prediction and complexity of mutation to protein function.

For non-functional mutations, another explanation is due to the existence of other stronger functional mutations, which veil the effects of other mutations. It is possible that mutations to one gene may provide cells with proliferation advantage but not enough for cancer, e.g., benign tumors. In this case, other functional mutations are necessary for further oncogenesis and expression patterns of cancer patients will mainly reflect the consequences of later mutations. Taking TP53 as an example, we observed that 107 breast cancer patients with functional TP53 mutations also carried 17 PIK3CA somatic mutations while 79 patients with non-functional TP53 mutations carried 27 PIK3CA somatic mutations. The significance of ratio differences was at p<0.018. This observation suggests that patients with functional TP53 mutation are less likely to carry PIK3CA mutations. If only considering the predicted functional PIK3CA mutations, no patient with functional TP53 mutations carried functional mutations to PIK3CA while 16 patients with non-functional TP53 mutations also carried functional mutations to PIK3CA. By checking other functional mutations, all 79 patients with non-functional TP53 mutations had at least one functional mutation to other genes. In summary, these results suggest non-functional TP53 mutations may result from the existence of other functional mutations.

Our method also faces the problem of sensitivity if not enough neighboring patients are available. Our method requires a minimum number of neighboring patients for enrichment analysis which is not always available. This is especially true for those mutations with low recurrence. With the progress of cancer genome sequencing projects, the number of patients with mutation information will increase, which will provide a better basis for functional mutation discovery.

Mutual exclusivity is one popular strategy used to recover driver mutations [15]. Based on the functional annotation with GO and KEGG terms, we checked this assumption with some well-known cancer genes. We observed success with mutual exclusivity in the patients with TP53, MAP3K1 and EGFR mutations. However, we also noticed one counter case with PIK3CA, which was involved in the PI3K signalling pathway [52]. Mutations to members of PI3K are more enriched in patients with PIK3CA mutations. This result suggests mutual exclusivity to be a good assumption in driver mutation discovery but also faces the possibility of failure.

## Figures and Tables

**Table 1:** Top 10 of functional mutations in breast cancer.

Mutated gene	No. somatic mutation	No. functional mutation	Percentage	p(MutSig)	p(MUSIC)	p(drGAP)
*PIK3CA*	175	123	70.3%	$7.47\times 10^{-12}$	0	$4.87\times 10^{-100}$
*TP53*	188	107	56.9%	0	0	$8.50\times 10^{-169}$
*MAP3K1*	40	30	75.0%	$3\times 10^{-7}$	0	$2.39\times 10^{-35}$
*CDH1*	35	29	82.6%	$8.73\times 10^{-9}$	0	$1.26\times 10^{-22}$
*GATA3*	56	19	33.9%	$9.8\times 10^{-12}$	0	$3.50\times 10^{-56}$
*RUNX1*	19	13	68.4%	1	$2.3\times 10^{-1}$	$2.8\times 10^{-1}$
*CTCF*	15	10	66.7%	1	1	$4.2\times 10^{-1}$
*CACNA1B*	14	9	64.2%	1	$5\times 10^{-2}$	$5.14\times 10^{-4}$
*DNAH17*	14	8	57.1%	1	$2.3\times 10^{-1}$	$2\times 10^{-2}$
*MAP2K4*	21	8	38.1%	$3.2\times 10^{-4}$	$6.0\times 10^{-4}$	$3.8\times 10^{11}$

## References

[1] Hanahan D., Weinberg R.A. (2000). The hallmarks of cancer. Cell.

[2] Cancer Genome Atlas Network (2012). Comprehensive molecular portraits of human breast tumours. Nature.

[3] Stephens P.J., Tarpey P.S., Davies H., Van Loo P., Greenman C., Wedge D.C., Nik-Zainal S., Martin S., Varela I., Bignell G.R. (2012). The landscape of cancer genes and mutational processes in breast cancer. Nature.

[4] Cancer Genome Atlas Network (2012). Comprehensive molecular characterization of human colon and rectal cancer. Nature.

[5] Vogelstein B., Papadopoulos N., Velculescu V.E., Zhou S., Diaz L.A., Kinzler K.W. (2013). Cancer genome landscapes. Science.

[6] Futreal P.A., Coin L., Marshall M., Down T., Hubbard T., Wooster R., Rahman N., Stratton M.R. (2004). A census of human cancer genes. Nat. Rev. Cancer.

[7] Stratton M.R., Campbell P.J., Futreal P.A. (2009). The cancer genome. Nature.

[8] Puente X.S., Pinyol M., Quesada V., Conde L., Ordóñez G.R., Villamor N., Escaramis G., Jares P., Beà S., González-Díaz M. (2011). Whole-genome sequencing identifies recurrent mutations in chronic lymphocytic leukaemia. Nature.

[9] Peifer M., Fernández-Cuesta L., Sos M.L., George J., Seidel D., Kasper L.H., Plenker D., Leenders F., Sun R., Zander T. (2012). Integrative genome analyses identify key somatic driver mutations of small-cell lung cancer. Nat. Genet..

[10] Hammerman P.S., Voet D., Lawrence M.S., Voet D., Jing R., Cibulskis K., Sivachenko A., Stojanov P., Mckenna A., Lander E.S. (2012). Comprehensive genomic characterization of squamous cell lung cancers. Nature.

[11] Gundem G., Perezllamas C., Jenesanz A., Kedzierska A., Islam A., Deupons J., Furney S.J., Lopezbigas N. (2010). IntOGen: Integration and data mining of multidimensional oncogenomic data. Nat. Methods.

[12] Banerji S., Cibulskis K., Rangel-Escareno C., Brown K.K., Carter S.L., Frederick A.M., Lawrence M.S., Sivachenko A.Y., Sougnez C., Zou L. (2012). Sequence analysis of mutations and translocations across breast cancer subtypes. Nature.

[13] Vandin F., Upfal E., Raphael B.J. (2012). Finding driver pathways in cancer: Models and algorithms. Algorithms Mol. Biol..

[14] Hadj Khodabakhshi A., Fejes A.P., Birol I., Jones S.J. (2013). Identifying cancer mutation targets across thousands of samples: MuteProc, a high throughput mutation analysis pipeline. BMC Bioinform..

[15] Ciriello G., Cerami E., Sander C., Schultz N. (2012). Mutual exclusivity analysis identifies oncogenic network modules. Genome Res..

[16] Ciriello G., Cerami E., Aksoy B.A., Sander C., Schultz N. (2013). Using MEMo to discover mutual exclusivity modules in cancer. Curr. Protoc. Bioinform..

[17] Vandin F., Upfal E., Raphael B.J. (2012). De novo discovery of mutated driver pathways in cancer. Genome Res..

[18] Leiserson M.D.M., Blokh D., Sharan R., Raphael B.J. (2013). Simultaneous identification of multiple driver pathways in cancer. PLoS Comput. Biol..

[19] Gonzalez-Perez A., Lopez-Bigas N. (2012). Functional impact bias reveals cancer drivers. Nucleic Acids Res..

[20] Leiserson M.D.M., Vandin F., Wu H., Dobson J.R., Eldridge J.V., Thomas J.L., Papoutsaki A., Kim Y., Niu B., Mclellan M.D. (2014). Pan-cancer network analysis identifies combinations of rare somatic mutations across pathways and protein complexes. Nat. Genet..

[21] Tran L.M., Zhang B., Zhang Z., Zhang C., Xie T., Lamb J.R., Dai H., Schadt E.E., Zhu J. (2011). Inferring causal genomic alterations in breast cancer using gene expression data. BMC Syst. Biol..

[22] Masica D.L., Karchin R. (2011). Correlation of somatic mutation and expression identifies genes important in human glioblastoma progression and survival. Cancer Res..

[23] Wiman K.G. (2010). Pharmacological reactivation of mutant p53: From protein structure to the cancer patient. Oncogene.

[24] Venselaar H., Te Beek T.A.H., Kuipers R.K.P., Hekkelman M.L., Vriend G. (2010). Protein structure analysis of mutations causing inheritable diseases. An e-Science approach with life scientist friendly interfaces. BMC Bioinform..

[25] Dehouck Y., Grosfils A., Folch B., Gilis D., Bogaerts P., Rooman M. (2009). Fast and accurate predictions of protein stability changes upon mutations using statistical potentials and neural networks: PoPMuSiC-2.0. Bioinformatics.

[26] Capriotti E., Fariselli P., Casadio R. (2005). I-Mutant2.0: Predicting stability changes upon mutation from the protein sequence or structure. Nucleic Acids Res..

[27] Johansson P., Pavey S., Hayward N. (2007). Confirmation of a BRAF mutation-associated gene expression signature in melanoma. Pigment Cell Res..

[28] Rebbeck T.R., DeMichele A., Tran T.V., Panossian S., Bunin G.R., Troxel A.B., Strom B.L. (2009). Hormone-dependent effects of FGFR2 and MAP3K1 in breast cancer susceptibility in a population-based sample of post-menopausal African-American and European-American women. Carcinogenesis.

[29] Lei H., Sjöberg-Margolin S., Salahshor S., Werelius B., Jandáková E., Hemminki K., Lindblom A., Vorechovský I. (2002). CDH1 mutations are present in both ductal and lobular breast cancer, but promoter allelic variants show no detectable breast cancer risk. Int. J. Cancer.

[30] Dees N.D., Zhang Q., Kandoth C., Wendl M.C., Schierding W., Koboldt D.C., Mooney T.B., Callaway M.B., Dooling D.J., Mardis E.R. (2012). MuSiC: Identifying mutational significance in cancer genomes. Genome Res..

[31] Hua X., Xu H., Yang Y., Zhu J., Liu P., Lu Y. (2013). DrGaP: A Powerful Tool for Identifying Driver Genes and Pathways in Cancer Sequencing Studies. Am. J. Hum. Genet..

[32] Janes K.A. (2011). RUNX1 and its understudied role in breast cancer. Cell Cycle.

[33] Van Bragt M., Hu X., Xie Y., Li Z. (2014). RUNX1, a transcription factor mutated in breast cancer, controls the fate of ER-positive mammary luminal cells. eLife.

[34] Docquier F., Farrar D., Darcy V., Chernukhin I., Robinson A.F., Loukinov D., Vatolin S., Pack S., Mackay A., Harris R.A. (2005). Heightened Expression of CTCF in Breast Cancer Cells Is Associated with Resistance to Apoptosis. Cancer Res..

[35] Labeit S., Kolmerer B. (1995). Titins: Giant proteins in charge of muscle ultrastructure and elasticity. Science.

[36] Opitz C.A., Kulke M., Leake M.C., Neagoe C., Hinssen H., Hajjar R.J., Linke W.A. (2003). Damped elastic recoil of the titin spring in myofibrils of human myocardium. Proc. Natl. Acad. Sci. USA.

[37] Brodie S.G., Deng C.X. (2001). BRCA1-associated tumorigenesis: What have we learned from knockout mice?. Trends Genet..

[38] Alberg A.J., Helzlsouer K.J. (1997). Epidemiology, prevention, and early detection of breast cancer. Curr. Opin. Oncol..

[39] Lu P., Weaver V.M., Werb Z. (2012). The extracellular matrix: A dynamic niche in cancer progression. J. Cell. Biol..

[40] Stetler-Stevenson W.G., Aznavoorian S., Liotta L.A. (1993). Tumor cell interactions with the extracellular matrix during invasion and metastasis. Annu. Rev. Cell Biol..

[41] Levine A.J. (1997). p53, the cellular gatekeeper for growth and division. Cell.

[42] Pearson G., Robinson F., Beers Gibson T., Xu B.E., Karandikar M., Berman K., Cobb M.H. (2001). Mitogen-activated protein (MAP) kinase pathways: Regulation and physiological functions. Endocr. Rev..

[43] Kennedy A.L., Morton J.P., Manoharan I., Nelson D.M., Jamieson N.B., Pawlikowski J.S., McBryan T., Doyle B., McKay C., Oien K.A. (2011). Activation of the PIK3CA/AKT pathway suppresses senescence induced by an activated RAS oncogene to promote tumorigenesis. Mol. Cell..

[44] Parsons D.W., Wang T.L., Samuels Y., Bardelli A., Cummins J.M., DeLong L., Silliman N., Ptak J., Szabo S., Willson J.K.V. (2005). Colorectal cancer: Mutations in a signalling pathway. Nature.

[45] Arroyo J.P., Kahle K.T., Gamba G. (2013). The SLC12 family of electroneutral cation-coupled chloride cotransporters. Mol. Asp. Med..

[46] Meng G., Vingron M. (2014). Condition-specific target prediction from motifs and expression. Bioinformatics.

[47] Langerød A., Zhao H., Borgan O., Nesland J.M., Bukholm I.R.K., Ikdahl T., Kåresen R., Børresen-Dale A.L., Jeffrey S.S. (2007). TP53 mutation status and gene expression profiles are powerful prognostic markers of breast cancer. Breast Cancer Res..

[48] Miller L.D., Smeds J., George J., Vega V.B., Vergara L., Ploner A., Pawitan Y., Hall P., Klaar S., Liu E.T. (2005). An expression signature for p53 status in human breast cancer predicts mutation status, transcriptional effects, and patient survival. Proc. Natl. Acad. Sci. USA.

[49] Lisowska K.M., Dudaladava V., Jarzab M., Huzarski T., Chmielik E., Stobiecka E., Lubinski J., Jarzab B. (2011). BRCA1-related gene signature in breast cancer: The role of ER status and molecular type. Front. Biosci. (Elite Ed.).

[50] Cizkova M., Cizeron-Clairac G., Vacher S., Susini A., Andrieu C., Lidereau R., Bièche I. (2010). Gene expression profiling reveals new aspects of PIK3CA mutation in ERalpha-positive breast cancer: Major implication of the Wnt signaling pathway. PLoS ONE.

[51] Dehouck Y., Kwasigroch J.M., Gilis D., Rooman M. (2011). PoPMuSiC 2.1: A web server for the estimation of protein stability changes upon mutation and sequence optimality. BMC Bioinform..

[52] Carnero A., Blanco-Aparicio C., Renner O., Link W., Leal J.F.M. (2008). The PTEN/PI3K/AKT signalling pathway in cancer, therapeutic implications. Curr. Cancer Drug Targets.

